# Innovative Flow Cytometry Allows Accurate Identification of Rare Circulating Cells Involved in Endothelial Dysfunction

**DOI:** 10.1371/journal.pone.0160153

**Published:** 2016-08-25

**Authors:** Federica Boraldi, Angelica Bartolomeo, Sara De Biasi, Stefania Orlando, Sonia Costa, Andrea Cossarizza, Daniela Quaglino

**Affiliations:** 1 Department of Life Sciences, University of Modena and Reggio Emilia, Via Campi 287, Modena, Italy; 2 Department of Surgery, Medicine, Dentistry and Morphological Sciences, University of Modena and Reggio Emilia, Via Campi 287, Modena, Italy; European Institute of Oncology, ITALY

## Abstract

**Introduction:**

Although rare, circulating endothelial and progenitor cells could be considered as markers of endothelial damage and repair potential, possibly predicting the severity of cardiovascular manifestations. A number of studies highlighted the role of these cells in age-related diseases, including those characterized by ectopic calcification. Nevertheless, their use in clinical practice is still controversial, mainly due to difficulties in finding reproducible and accurate methods for their determination.

**Methods:**

Circulating mature cells (CMC, CD45^-^, CD34^+^, CD133^-^) and circulating progenitor cells (CPC, CD45^dim^, CD34^bright^, CD133^+^) were investigated by polychromatic high-speed flow cytometry to detect the expression of endothelial (CD309^+^) or osteogenic (BAP^+^) differentiation markers in healthy subjects and in patients affected by peripheral vascular manifestations associated with ectopic calcification.

**Results:**

This study shows that: 1) polychromatic flow cytometry represents a valuable tool to accurately identify rare cells; 2) the balance of CD309^+^ on CMC/CD309^+^ on CPC is altered in patients affected by peripheral vascular manifestations, suggesting the occurrence of vascular damage and low repair potential; 3) the increase of circulating cells exhibiting a shift towards an osteoblast-like phenotype (BAP^+^) is observed in the presence of ectopic calcification.

**Conclusion:**

Differences between healthy subjects and patients with ectopic calcification indicate that this approach may be useful to better evaluate endothelial dysfunction in a clinical context.

## Introduction

Endothelial dysfunction is instrumental in the development and progression of many cardiovascular disorders. Therefore, the possibility of using non-invasive techniques to evaluate endothelium damage and repair potential has a relevant clinical value. Circulating endothelial cells consist of mature endothelial cells detaching from the intima monolayer in response to endothelial damages [1]. These cells are detectable in peripheral blood. Indeed, even if are rare in healthy individuals, they can be more abundantly detected in patients with cardiovascular-related complications [2–4], suggesting that they may be taken as indicator of disease severity [5].

When injury or tissue damage occurs, circulating progenitor cells are thought to mobilize from bone marrow into the circulation, homing to sites of tissue repair under the guidance of several signals [6]. To assure an adequate homeostatic tissue control, repair activities should compensate the extent of damage processes, if not, endothelial dysfunction takes place. Moreover, endothelial and vascular smooth muscle cells can differentiate into osteoblast-type cells [7], thus contributing to ectopic calcification, one of the most frequent complication in the aging vasculature. These cells might originate from resident vascular mesenchymal progenitors, from trans-differentiation of mature vascular smooth muscle cells or from circulating cells with a calcifying potential [8,9].

Despite the number of studies performed so far, investigation on circulating endothelial and progenitor cells is technically challenging and contradictory results are frequently reported, due to discrepancies in terms of terminology and protocols used for the detection of these cells, thus leading to ambiguous conclusions affecting the significance of data in the clinical practice [10].

The present study has been undertaken with the aim to investigate circulating cells in healthy subjects and in patients affected by peripheral vascular manifestation associated with ectopic calcification. Thus, circulating mature cells (CMC, CD45^-^, CD34^+^, CD133^-^) and circulating progenitor cells (CPC, CD45^dim^, CD34^bright^, CD133^+^) were firstly identified by polychromatic high-speed flow cytometry and on both CPC and CMC, markers of endothelial (CD309) or osteogenic (bone alkaline phosphatase, BAP) differentiation were analysed.

As a model, we used blood from patients affected by *Pseudoxanthoma elasticum*, a genetic disorder characterized by the premature occurrence of *claudication intermittens* and by a progressive mineralization of elastic fibres within soft connective tissues [11,12]. This approach allows evaluating if the proposed methodology is capable to highlight differences between individuals without any clinical evidence of vascular manifestations and patients affected by peripheral artery complications. Furthermore, since these patients are characterized by ectopic calcification, we investigated if these circulating cells exhibit a shift towards an osteoblast-like phenotype.

## Materials and Methods

### Blood sample collection

Up to 33 mL peripheral blood were collected in EDTA-coated tubes from 20 patients affected by *Pseudoxanthoma elasticum* (PXE) (mean age± std: 44±16 yr) and from 22 healthy subjects (47±15 yr). Patients suffered from vascular alterations (*claudication intermittens*, hypertension). Clinical diagnosis of PXE was molecularly confirmed by demonstrating two causative mutations in the ABCC6 gene. This study was performed in accordance with the guidelines of the Helsinki declaration and approved by the Medical Ethical Committee of the University of Modena and Reggio Emilia (#35/15). All individuals gave signed informed consent.

Samples were processed immediately after venepuncture. In order to avoid the interference of endothelial cells damaged by needle insertion through the vessel wall, the first 3 mL of blood were discarded [5]. Peripheral blood cells were obtained according to standard protocols. For the evaluation of circulating cytokines and growth factors, plasma was centrifuged at 3,500 rpm for 15 min at 4°C and stored immediately at -80°C until analyses.

### Flow cytometry analysis

**Fig 1:**
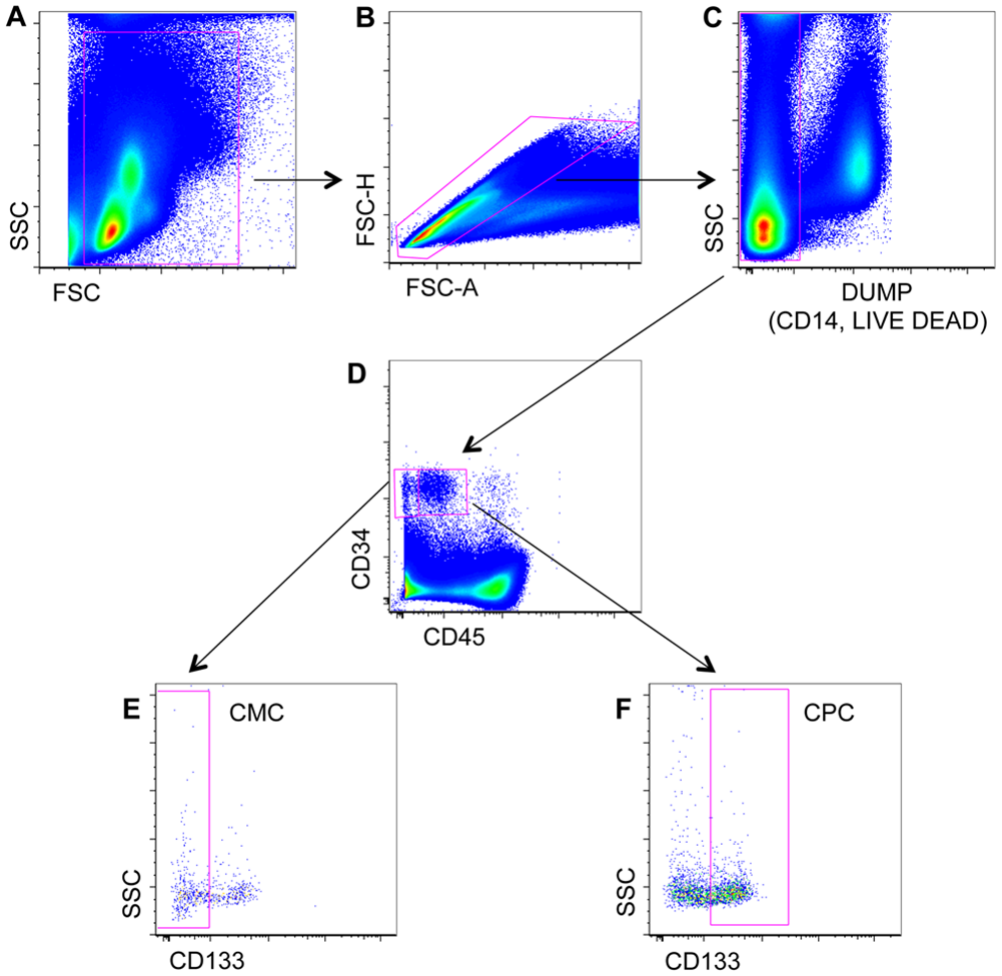
Gating strategy for the identification of circulating progenitor cells (CPC, CD45^dim^, CD34^bright^, CD133^+^) and circulating mature cells (CMC, CD45^-^, CD34^+^, CD133^-^). (A-B) Peripheral blood mononuclear cells were gated according to physical parameters. (C) Debris, monocytes and dead cells were removed by using an electronic gate and the dump channel (containing mAbs against CD14 and a viability marker, i.e. Live Dead). CMC and CPC were identified on the basis of the expression of CD34, CD45 and CD133. (D-E) CMC were defined as CD45^-^, CD34^+^, CD133^-^. (D-F) CPC were defined as CD45^dim^, CD34^bright^, CD133^+^.

Circulating mature cells (CMC, CD45^-^, CD34^+^, CD133^-^) and circulating progenitor cells (CPC, CD45^dim^, CD34^bright^, CD133^+^) were evaluated by 2-laser flow cytometry using a panel of monoclonal antibodies, including those recognizing monocyte/macrophage marker CD14-APCH7 (Becton Dickinson, Milan, Italy–BD), Live Dead far red (Life Technologies—Thermo Fisher Scientific), leukocyte common antigen CD45-PE (R&D System, Minneapolis, MN, USA), hematopoietic progenitor cell antigen CD34-PC7 (Beckman Coulter, Milan, Italy), stem cell marker CD133-APC (Miltenyi, Bologna, Italy), endothelial differentiation marker CD309-FITC (R&D System) or osteogenic marker bone alkaline phosphatase BAP-FITC (R&D System). Cells were first gated on the basis of forward scatter (FSC) and side scatter (SSC). Doublets were removed by physical parameters. Dead cells, B cells, monocytes and cell debris were removed by the use of electronic gate and the dump channel (containing mAbs against CD14 and Live Dead). A functional hierarchy can be performed based for instance on the progressive expression of differentiation surface markers (i.e. negative, dim, positive, bright). At this point circulating mature and progenitors cells were defined as CD45^-^/CD34^+^/CD133^-^ and CD45^dim^/CD34^bright^/CD133^+^, respectively (Fig 1). These cells were finally analysed for the expression of CD309 or BAP.

Using a novel strategy for the identification of rare events [13], a minimum of 5 million cells per sample was acquired using an 8-parameters Attune Acustic Focusing Flow Cytometer (Thermo Fisher Scientific), equipped with lasers at 488 nm and 634 nm. Data regarding CPC and CMC were then analysed by FlowJo 9.9.3 (Treestar Inc., Ashland, OR) under MacOS 10 [14]. Single staining and Fluorescence Minus One (FMO) controls were performed for all panels to set proper compensation and define positive signals [15].

### Quantification of soluble molecules

Plasma levels of vascular endothelial growth factor (VEGF), stromal-derived factor-1alpha (SDF-1α); Interleukin-1beta (IL-1β); interleukin-6 (IL-6); tumor necrosis factor-alpha (TNF-α) and soluble receptor for advanced glycosylated end products (sRAGE) were determined using commercial ELISA kit according to manufacturer’s instructions (Quantikine—R&D Systems). Measurements were done in triplicate.

### Statistical analysis

Statistical analysis was performed using GraphPad Prism software, version 5.01 for MAC (GraphPad Software, San Diego, CA, USA). Comparison between results obtained in healthy subjects and in patients was performed using the Shapiro-Wilk and Mann–Whitney tests. P values less than 0.05 were considered statistically significant.

## Results

### CMC from patients with vascular manifestations show an increased expression of BAP

**Fig 2:**
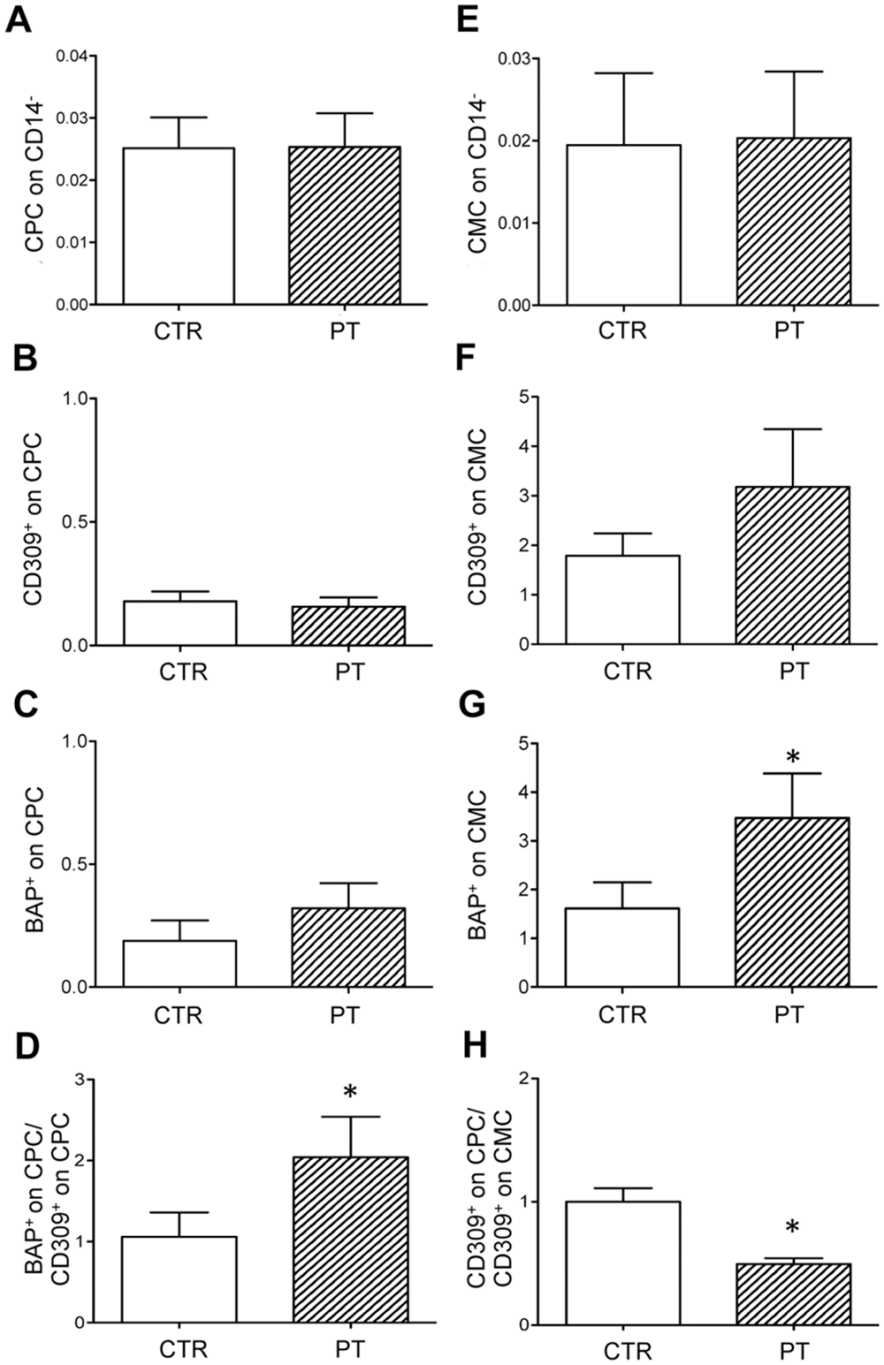
Phenotypic characterization of circulating progenitor cells (CPC, CD45^dim^, CD34^bright^, CD133^+^) and circulating mature cells (CMC, CD45^-^, CD34^+^, CD133^-^) in healthy subjects (CTR) and in patients (PT). (A,E) The percentage of CPC and CMC on CD14^-^ is similar in CTR and in PT. (B-C) The percentage of CD309^+^ (marker of endothelial differentiation) or BAP^+^ (osteogenic marker) cells on CPC in CTR and in PT. (D) The different BAP^+^/CD309^+^ ratio on CPC suggests that circulating progenitor cells in patients undergo a shift towards an osteogenic phenotype. (F-G) Histograms show the percentage of CD309^+^ or BAP^+^ on CMC in CTR and in PT. (H) The different CD309^+^ on CPC/CD309^+^ on CMC ratio suggests that patients have a lower vascular repair potential. Values are shown as mean ± SD. *p<0.05.

Circulating progenitor cells (CPC, CD45^dim^/CD34^bright^/CD133^+^) and circulating mature cells (CMC, CD45^-^/CD34^+^/CD133^-^) were identified by a highly sensitive acoustic flow cytometer capable to analyse up to 35,000 cells per second. The percentage of these circulating cell populations was similarly represented in patients and in control subjects (Fig 2A and 2E). Therefore, starting from a comparable percentage of cells, we have assessed the presence of the endothelial marker of differentiation CD309 and of the osteogenic marker BAP.

The percentage of CD309^+^ cells and of BAP^+^ cells on CPC in healthy subjects and in patients was similar (Fig 2B and 2C). However, the ratio of BAP^+^/CD309^+^, taken as an indicator of the phenotypic shift of CPC towards an osteoblast-like phenotype, highlighted a two-fold increase in patients versus healthy subjects (Fig 2D).

We also investigated CMC positive for CD309 or for BAP. The percentage of CD309^+^ cells was generally higher in patients in comparison to healthy subjects (Fig 2F), but the ratio between CD309^+^ on CPC and CD309^+^ on CMC was significantly lower in patients than in healthy subjects (Fig 2H). Interestingly, the increased percentage of BAP^+^ cells detected in patients demonstrates in these individuals the presence of an osteoblast-like phenotypic shift (Fig 2G).

### Patients with vascular manifestations showed increased plasma levels of VEGF

**Fig 3:**
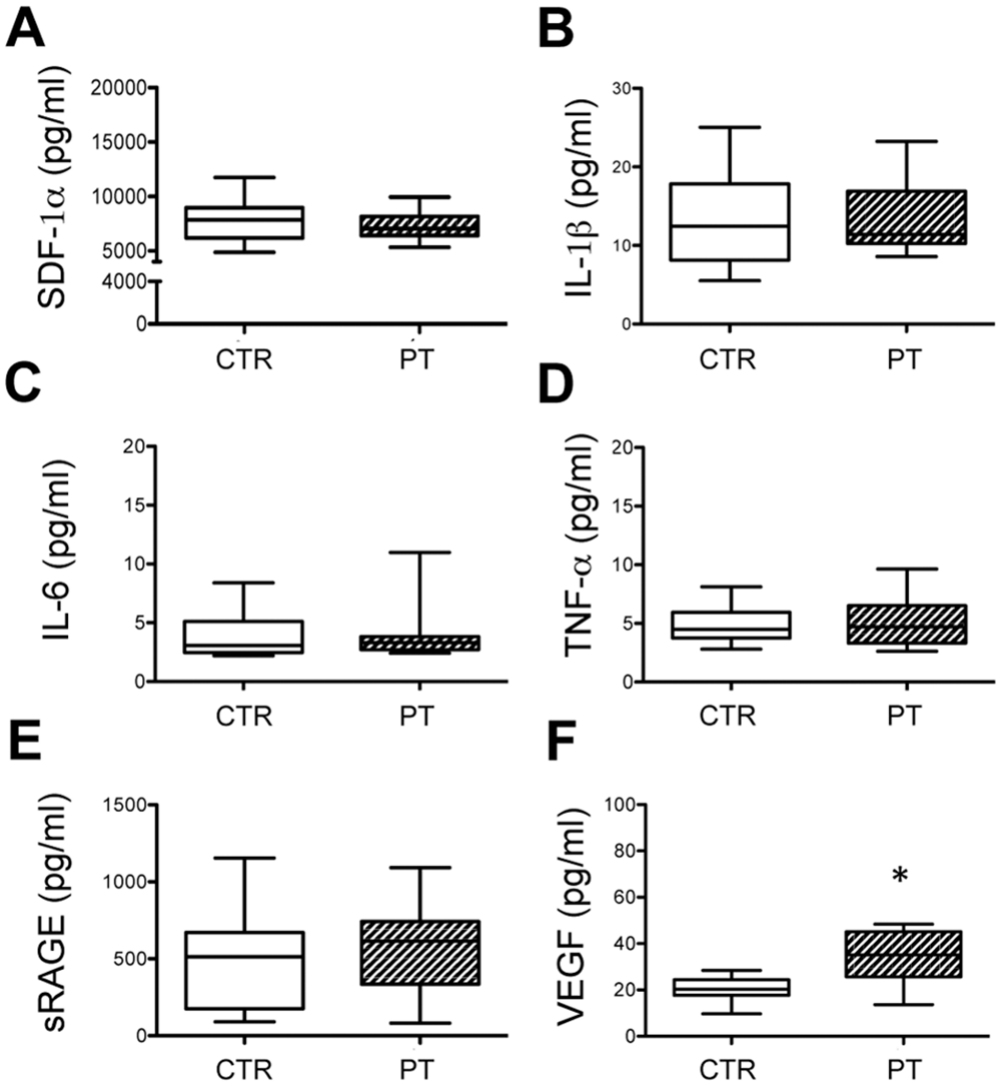
ELISA test. Amount of stromal-derived factor-1alpha (SDF-1α) (A); interleukin-1beta (IL-1β) (B); interleukin-6 (IL-6) (C); tumour necrosis factor-alpha (TNF-α) (D); soluble receptor for advanced glycosylated end products (sRAGE) (E) and vascular endothelial growth factor (VEGF) (F) were determined in plasma of healthy subjects (CTR) and of patients (PT). Values are shown as mean ± SD. *p<0.05.

In order to evaluate if vascular damage is related to inflammatory markers, cytokines (IL-1β, IL-6 and TNF-α growth factors (SDF-1α and VEGF) as well as the soluble factor sRAGE were quantified in plasma from controls and from patients. VEGF levels were significantly higher in patients than in healthy subjects (Fig 3F), whereas no significant differences were observed as far as SDF-1α IL-1β, IL-6, TNF-α and sRAGE expression (Fig 3A–3E).

## Discussion

Several approaches have been used to identify circulating rare cells and, even though flow cytometry appears as the most promising and rapid technique, no consistent and conclusive data are reported so far, mainly because of the limited number of antigens detected as well as their expression on overlapping phenotypes [16]. It has to be underlined, indeed, that the identification of circulating rare cells (i.e., those representing 0.0001 to 0.01% of peripheral blood cells), requires the acquisition of a large number of events, typically of the order of several millions, and that the amount of some antigens on the cell surface can be so low that conventional flow cytometers are not able to identify “dim” and “bright” populations according to the intensity of fluorescence of a given antigen-bound antibody.

To cope with the aforementioned problems, the cytometer used in the present study is the first one that applies ultrasonic waves (over 2 MHz, similar to those used in medical imaging) rather than hydrodynamic forces to position cells into a single focused line along the central axis of a capillary. Keeping cells within a confined focal point is a crucial requirement for the consistent excitation of conjugated fluorochromes, for maintaining the same sample speed at all flow rates, and especially for reaching an incredible speed of acquisition. This approach allows a relatively easy analysis of rare events, with a speed that is about 50–100 times higher than that typically used in this field. Finally, it has to be underlined that, from a statistical point of view, the analysis of a dramatically high number of events is crucial to obtain the accuracy that is required for the identification and quantification of rare cells. Based on this new technology and on the knowledge on specific marker studies [17], we were able to accurately identify cells discriminating between circulating progenitor cells (CPC, CD45^dim^/CD34^bright^/CD133^+^) and circulating mature cells (CMC, CD45^-^/CD34^+^/CD133^-^).

No changes were observed in the number of CPC in controls and in patients. These data are in agreement with the similar amount of plasma SDF-1α growth factor responsible for the mobilization of stem and progenitor cells from bone marrow to blood [18].

Circulating mature endothelial cells detached from the intima of the vessel wall (CD309^+^ on CMC) are considered markers of endothelial damage [2,4]. Interestingly, their number, although not statistically significant compared to controls, exhibits an increased trend in patients with vascular clinical manifestations. Moreover, the ratio between two different pool of circulating endothelial-positive cells has been previously suggested to be a measure of vascular health [19–20], because CD309^+^ on CPC inform on the endothelial repair capacity [21], whereas CD309^+^ on CMC are indicators of ongoing endothelial damage [2]. The strong decrease of this ratio in patients may therefore indicate that in these subjects there is a reduced vascular repair potential indicative of endothelial dysfunction.

In a number of diseases (i.e. diabetes, kidney disease, atherosclerosis and coronary artery diseases) endothelial damages are also associated with vascular calcification [22]. It has been demonstrated that procalcific polarization of circulating progenitors may contribute to vascular mineralization in patients exhibiting a calcifying potential in vitro [9,23]. The ratio of bone (BAP) versus endothelial (CD309) marker expression highlights that, in patients with ectopic calcification, CPC undergo a shift towards an osteogenic phenotype. It could be suggested that, when progenitor cells are recruited to sites of vascular damage, they could promote vascular calcification. Moreover, in patients’ peripheral blood, an increase of BAP^+^ on CMC was also found. Ectopic calcification is a frequent complication of aging vessels, significantly contributing to cardiovascular manifestations [24]. Therefore, the possibility to evaluate the osteogenic phenotypic shift of circulating cells [23], in addition to measurement of vascular health [19–20], represents a valuable non-invasive tool for a better management of the aging population at increased risk of cardiovascular events.

It is also known that angiogenesis contributes to aberrant mineralization since new vessels can act as a conduit for osteo-progenitor cells including both circulating progenitor cells and pericytes present within vessels [7]. The increased amount of plasma VEGF measured in patients characterized by ectopic calcification suggests that VEGF, favoring the angiogenic process, may contribute to ectopic calcification by recruiting osteoblast-like cells at specific sites.

In order to exclude that calcification in these patients is the consequence of a generalized inflammatory process, we measured the amount of inflammatory markers as IL-1β, IL-6, TGF-β, TNF-α and s-RAGE [25–27]. No changes were detected for these soluble factors, further demonstrating that ectopic calcification may take place also in the absence of an inflammatory condition.

## Conclusions

Although limited by the relatively small number of subjects, this study represents a proof of concept that: 1) the use of new-generation of polychromatic high speed flow cytometry is crucial to accurately identify rare cells; 2) altered CD309^+^ on CMC/CD309^+^ on CPC balance, suggestive of vascular damage and low repair potential, can be revealed in patients with disease-affected peripheral vessels also in the absence of a clinically relevant inflammatory condition; 3) an increase of circulating cells with a shift towards an osteoblast-like phenotype might be related to the presence of ectopic calcification. Thus, our results may pave the way to future studies on a larger cohort of individuals for the potential use of this approach to better evaluate endothelial dysfunction in a clinical context.
